# Heavy Metals in the Soil–Coffee System of Pu’er, China, a Major Coffee Producing Region in China: Distribution and Health Risks

**DOI:** 10.3390/toxics13110944

**Published:** 2025-11-01

**Authors:** Xiaohua Zhou, Tianyao Yang, Yupei Hao, Jing Li, Bai Du, Sheping Yang, Xiongyi Miao

**Affiliations:** 1 Yunnan Provincial Bureau of Geology and Mineral Exploration and Development Center Laboratory, Key Laboratory of Sanjiang Metallogeny and Resources Exploration and Utilization Ministry of Natural Resources, Yunnan Key Laboratory of Sanjiang Metallogeny and Resources Exploration and Utilization, Yunnan Engineering Research Center of Exploration and Comprehensive Utilization of Mineral Resources, Kunming 650051, China; zhouxiaohua202408@126.com (X.Z.); lj13577110934@126.com (J.L.); 13708881943@139.com (B.D.); y7211177@126.com (S.Y.); 2 School of Geography and Environmental Science, School of Karst Science, Guizhou Normal University, Guiyang 550001, China; yangty515@163.com; 3 Department of Modern Engineering, Anshun Technical College, Anshun 561000, China

**Keywords:** heavy metal distribution, soil–coffee system, health risk assessment, coffee plants

## Abstract

This study provides a comprehensive assessment of the distribution, bioaccumulation, and health risks associated with heavy metals in the soil–coffee system of Pu’er City, a major coffee-producing region in China. An analysis of the soil and corresponding plant samples (including fruit, stem, and leaf) from representative plantations revealed that, although the heavy metal concentrations in soils generally exceeded the local background levels, they remained below national risk screening thresholds. Hg was identified as the primary pollutant of concern, showing moderate to significant enrichment (EF: 2–20) and posing a moderate to considerable ecological risk (E_i_: 40–160). In coffee plants, most heavy metals accumulated predominantly in the stems, whereas Pb and As were more concentrated in the leaves and fruits, respectively. Among the studied metals, only Cu exhibited a notable bioconversion tendency, with a biota soil accumulation factor (BSAF) close to 1, while other metals showed limited transfer (BSAF < 1). A generally negative correlation was observed between the soil metal content and BSAF, suggesting that elevated total concentrations do not necessarily enhance bioavailability. The health risk assessment indicated that coffee consumption poses no significant non-carcinogenic risk (HI < 1). However, the carcinogenic risks for Cr and As, albeit within acceptable limits (LCR between 10^−6^ and 10^−4^), still warrant attention. These findings underscore the importance of implementing targeted source control for Hg and Cr in soils and further investigating the transfer mechanisms of As to support the sustainable and safe production of coffee in this region.

## 1. Introduction

The contamination of heavy metals in soil has emerged as a pervasive global environmental issue [1,2,3,4], posing a significant threat to the security of the agricultural ecosystem and various foods [5,6]. The excessive accumulation of heavy metals in agricultural soils, derived from both the natural geological background and anthropogenic activities such as mining [7], irrigation [1] with contaminated water, and the application of fertilizers [8] and pesticides [9], can detrimentally affect crop growth, yields, and quality [10,11,12]. More critically, heavy metals in soil possess the characteristics of persistence, bioaccumulation, and toxicity [13,14,15,16]. They can be absorbed by crops and subsequently transferred through the food chain, ultimately posing potential carcinogenic and non-carcinogenic health risks to human consumers [17,18,19]. Consequently, the distribution, bioavailability, and health risks of heavy metals in soil–crop systems have been extensively investigated [14,20,21]. While considerable research has focused on staple crops with high consumption rates, such as rice [22], wheat [23], and vegetables [12], significantly less attention has been paid to economic crops with relatively limited dietary intake.

Coffee is renowned worldwide for its rich content of caffeine, chlorogenic acid, various antioxidants [24,25], etc. Consuming coffee can not only mitigate fatigue by mildly stimulating the central nervous system but also protect against diseases associated with inflammation and oxidative stress, such as obesity, metabolic syndrome, and type 2 diabetes [26,27]. Following its discovery, coffee rapidly spread across the world and has become one of the most popular beverages globally [26]. Driven by substantial international demand, coffee cultivation has expanded significantly, emerging as an economic cornerstone in many subtropical and tropical regions [28,29,30]. In China, coffee was first introduced in Hainan; however, the more favorable climate enabled Yunnan Province to surpass Hainan Province to become the predominant coffee-growing region [25]. According to earlier reports, China’s coffee production in 2024 reached approximately 146,000 tons, with Yunnan Province alone accounting for over 98% of the total output, solidifying its role as the nation’s leading production base for coffee [25,31]. The region primarily cultivates high-quality Arabica coffee, which thrives in tropical and subtropical mountainous areas. Consequently, cities such as Pu’er, Xishuangbanna, and Lincang in Southern Yunnan Province have become key hubs for coffee cultivation.

These areas are generally characterized by high geological background levels of heavy metals [32,33]. In addition to the inherently elevated heavy metal concentrations in the soil, the local warm, humid, and rainy climate contributes to acidic soil conditions [31,34]. It is well established that soil acidity significantly enhances the bioavailability and mobility of heavy metals [35,36], facilitating their uptake by plants. Although coffee beans undergo roasting and brewing before consumption—processes that may alter the heavy metal exposure risks compared to those associated with leafy vegetables or grain crops—this does not imply that the coffee production system is inherently immune to the contamination of heavy metals. The stresses caused by heavy metals can not only impair the physiological health and yields of coffee plants [37] but also lead to their accumulation in the edible beans, directly threatening beverage safety and market credibility. Given the current lack of comprehensive studies on heavy metal enrichment in coffee produced in Yunnan Province, there is an urgent need to conduct relevant investigations to clarify the health risks of coffee consumption in relation to exposure to heavy metals.

Pu’er City is recognized as the coffee capital of China, accounting for nearly half of both the cultivation area and production in Yunnan Province over the long term. The coffee industry plays a pivotal role in driving regional economic development, which is why Yunnan Province has prioritized the dual improvement of its coffee output and quality in Pu’er City as a core objective for local industry growth [38]. While advancements in coffee cultivation techniques can enhance both yields and quality, expanding the scale of eco-friendly coffee farming is likely to have an even greater synergistic effect. However, given the naturally high background levels of heavy metals in the soils of Pu’er City [34], the indiscriminate expansion of coffee plantations may not only fail to improve yields and quality but could also compromise bean quality and increase the health risks associated with contaminated coffee consumption. Therefore, to support the scientifically informed zoning and sustainable scaling of coffee cultivation in Pu’er City, a comprehensive investigation into the pollution caused by heavy metals in the soil–coffee system is essential. Such efforts are crucial in enhancing the reputation of Pu’er City in the global coffee market and establishing the region as a reliable source of high-quality coffee beans. The heavy metals targeted in this study—chromium (Cr), copper (Cu), zinc (Zn), lead (Pb), cadmium (Cd), arsenic (As), and mercury (Hg)—were selected as they are priority contaminants regulated by China’s environmental quality standards for agricultural soils (GB 15618-2018) and are of global concern due to their persistence, toxicity, and potential for bioaccumulation [1,13,16]. Given the current lack of comprehensive studies on heavy metal enrichment in coffee produced in Yunnan Province, there is an urgent need to conduct relevant investigations. Accordingly, this study aimed to (1) determine the concentrations of heavy metals in the soils of Pu’er City; (2) elucidate the distribution and bioaccumulation of heavy metals in various parts of the coffee plant, including the beans, leaves, and stems; and (3) evaluate the health risks posed by heavy metal exposure through coffee consumption. The results are expected to provide a scientific foundation for soil environmental zoning and risk management strategies in coffee-growing regions.

## 2. Materials and Methods

### 2.1. Description of the Study Area and Field Sampling

Pu’er City is situated in Southwestern Yunnan Province (22°02′–24°50′ N, 99°09′–102°19′ E), on the edge of the Yunnan–Guizhou Plateau, and falls within a typical subtropical plateau monsoon climate zone. The region has a mean annual temperature of 15–20 °C and receives abundant rainfall, with average annual precipitation of approximately 1500 mm, characterized by distinct alternating wet and dry seasons. The terrain is predominantly mountainous and hilly, with elevation of 800~1500 m. The coffee plantations in Pu’er City are predominantly established on highly weathered, acidic soils (Acrisols and Ferralsols), all of which are characterized by the accumulation of iron and aluminum oxides, which impart a distinct reddish color [38,39]. The soil pH ranges from 4.5 to 6.5, aligning with the advanced weathering stage. The parent materials primarily consist of Quaternary residual-slope deposits and alluvial–proluvial deposits. The surface layer (0–10 cm) is typically rich in organic matter (often forming an umbric or ochric horizon), underlain by an argic horizon (Acrisols) or a ferralic horizon (Ferralsols) with a strong granular structure, which promotes good drainage and aeration and facilitates deep root penetration.

**Figure 1:**
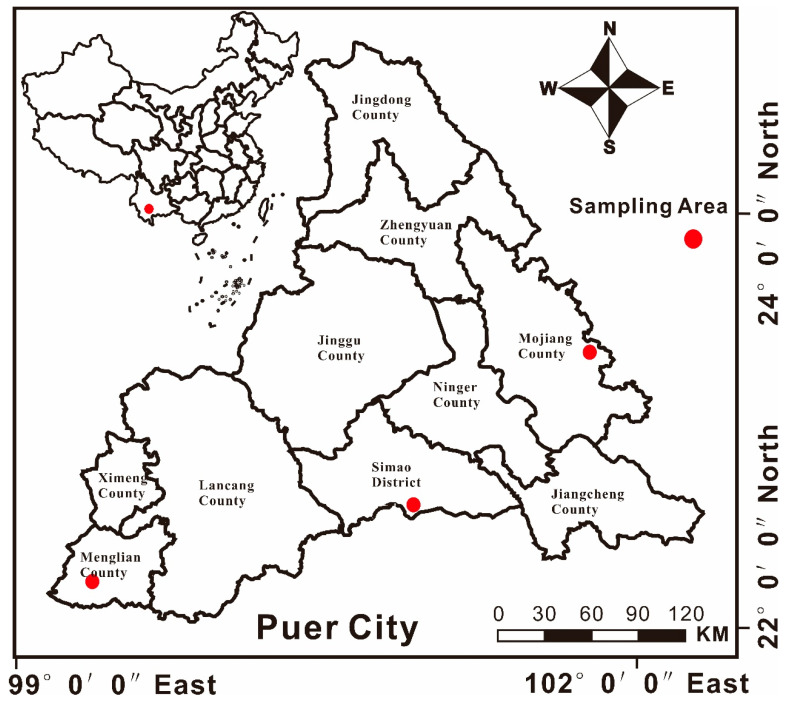
Study area in Pu’er City, China.

The area also exhibits naturally high background levels of heavy metals such as Cr, Pb, and As [38,39]. Owing to its unique topographic and climatic conditions, Pu’er City has become one of the most important coffee-growing regions in Yunnan Province, earning the title of “China’s Coffee Capital” [38]. The primary coffee variety cultivated here is *Coffea arabica* L., with major plantations concentrated in Simao County, Menglian County, and Mojiang County. Coffee cultivation in these areas has become a pillar of the local agricultural economy, significantly contributing to farmers’ incomes and regional development. To accurately reflect the distribution of heavy metals in the soil–coffee system, sampling for this study was conducted mainly in the counties of Simao, Menglian, and Mojiang, with widespread coffee plantations (see Figure 1). Since the coffee ripening period in Yunnan Province spans from late November to February of the following year, sample collection was carried out in January–February 2024. To comprehensively assess the distribution of heavy metals in coffee, a total of 30 sampling sites were established across multiple plantations in Simao County, Menglian County, and Mojiang County, with 10 sites in each county. At each site, both soil and coffee plant samples were collected. For plant sampling, leaves, stems, and fresh fruits were taken, yielding a total of 90 plant samples. The coffee bean is the edible fruit of the coffee plant, so it was collected as the coffee fruit. Following plant collection, topsoil (0–10 cm depth) samples were gathered from three mixed subsamples near each plant, resulting in 30 composite soil samples. All samples were bagged and transported to the laboratory for pretreatment.

### 2.2. Sample Preparation and Analysis

Debris from roots and rocks was screened out from the soils; then, the samples of soil were air-dried naturally, ground, and passed through a 0.149 mm nylon sieve before storage. Plant samples were rinsed with tap water, followed by washing with deionized water; then, they were deactivated at 105 °C for 30 min and dried to a constant weight at 75 °C before being ground and stored. For the determination of the total heavy metals in soils, a strong acid digestion method was employed. Briefly, 0.5 g of the finely ground soil sample was digested with a mixture of 9 mL HNO_3_ and 3 mL HCl in a microwave digestion system. The digestion program was as follows: ramped to 120 °C in 10 min and held for 5 min and then further ramped to 180 °C in 10 min and held for 20 min. After cooling, the digestate was filtered, transferred to a 50 mL volumetric flask, and made up to volume with deionized water. For plant sample digestion, 0.5 g of each sample was placed in a digestion vessel, to which 5 mL of HNO_3_ and 2 mL of H_2_O_2_ were added. The vessels were sealed and digested using a microwave digestion system. Heavy metals in soil samples were quantified by graphite furnace atomic absorption spectrometry (Shimadzu AA6880, Shimadzu Corporation, Kyoto, Japan). The heavy metal content in plant tissues was determined by inductively coupled plasma mass spectrometry (ICP-MS, iCAP RQ, Thermo Fisher Scientific, Waltham, MA, USA).

### 2.3. Quality Assurance and Quality Control

Quality assurance and quality control (QA/QC) were implemented for each batch of samples, which included the analysis of procedural blanks and certified reference materials (CRMs) for every ten samples. Quality control was performed using certified reference material GBW 07405 throughout the analytical procedure. The CRMs were obtained from the Chinese Academy of Measurement Sciences. All measured recovery rates fell within the acceptable range of 95–105%. The limits of detection (LODs) and limits of quantitation (LOQs) for each heavy metal were, respectively, 0.01 mg/kg and 0.03 mg/kg for As, 0.001 mg/kg and 0.003 mg/kg for Hg, 0.005 mg/kg and 0.015 mg/kg for Cd, 0.02 mg/kg and 0.06 mg/kg for Pb, 0.05 mg/kg and 0.15 mg/kg for Cr, 0.03 mg/kg and 0.09 mg/kg for Cu, and 0.10 mg/kg and 0.30 mg/kg for Zn [17].

### 2.4. Enrichment Factor Index

The non-dimensional enrichment factor (EF) is widely used to assess the contamination status of heavy metals and to identify their potential sources (e.g., natural or anthropogenic) [40,41]. Following established methodologies, aluminum (Al) was selected as the normalizing element to account for the influence of the grain size on heavy metal concentrations [41]. The calculation of the EF is based on the following equation:

$$EF=\frac{(\frac{C_{x}}{C_{Al}})sample}{(\frac{Cx}{CAl})baseline}$$

where (C_x_/C_Al_) is the ratio of metal to Al for the measured sample and in the reported baseline, respectively. The baseline used in calculation referred to the upper continental crust of North China [42]. The classification of heavy metal enrichment can be found in Table 1.

### 2.5. Potential Ecological Risk Index for Heavy Metals

The potential ecological risk index (RI) is proposed to evaluate the ecological risks of toxic substances and pollutants to biotas [43,46,47,48]. The formulas for RI calculation are shown below:

$$C_{f}^{i}=\frac{C^{i}}{C_{n}^{i}}\;E_{r}^{i}=T_{r}^{i}\times C_{f}^{i}\;RI=\sum_{i=1}^{n}E_{r}^{i}$$

where $C^{i}$ represents the concentration of heavy metal i in soil, mg/kg; $C_{n}^{i}$ is the relevant background value of heavy metals in the soil of Yunnan Province; and $T_{r}^{i}$ is the biological toxicity factor of each heavy metal. The values of $C_{n}^{i}$ and $T_{r}^{i}$ can be found in Table 2. $C_{f}^{i}$ is the single-element contamination factor, which is the ratio between $C^{i}$ and $C_{n}^{i}$. $E_{r}^{i}$ and RI are, respectively, the monomial ecological risk factor of an individual heavy metal and the total ecological risk index, see Table 3. The grades for ecological risk assessment are summarized in Table 1.

### 2.6. Calculation of Heavy Metal Bioconversion

The biota–soil accumulation factor (BSAF) is defined as the ratio of the heavy metal concentration in the biota (coffee) to its concentration in the soil. This ratio reflects the bioaccumulation potential of contaminants and is therefore commonly applied to assess the bioconversion of various pollutants [50,51]. It is calculated using the following equation:

BSAF = C_biota_/C_soil_

C_biota_ and C_soil_ (dry weight mg/kg) are the concentrations of heavy metals in the coffee fruit and in soils, respectively. A higher BSAF value indicates the greater bioaccumulation of heavy metals by organisms from soils and the enhanced bioconversion of heavy metals in soils. When BSAF > 1, the element tends to accumulate in organisms; conversely, when BSAF < 1, the element tends to remain in the soil, with limited bioconversion.

### 2.7. Assessment of Health Risk of HMs in Coffee Fruit

The health risks of coffee fruit associated with heavy metal exposure were evaluated in this study. As the most popular drink across the world, coffee fruits are commonly powdered for drink making, but the ingestion of the coffee fruit in drinks is only one way in which heavy metals threaten public health. The method of health risk assessment was proposed by the USEPA [52], which includes the evaluation of the probable non-carcinogenic and carcinogenic risks of heavy metal exposure from ingestion. In the assessment of health risks, the consumption rate of coffee fruit is a critical parameter that should be determined, considering the frequency of coffee consumption and the corresponding usage amount in a typical cup. In both coffee industry standards and culinary guidelines, the amount of coffee beans commonly required to brew a standard serving falls between 7 and 15 g, the average of which is 10 g/cup [53,54]. Applying daily one-cup consumption would likely overestimate the long-term risk of coffee consumption, as not all consumers in China drink coffee daily. Industry data, such as those from the China Coffee Consumption Market Report in 2021, indicate that, even among Chinese coffee drinkers, the consumption frequency of coffee is generally 3 to 5 times per week, the median value of which is 4 times per week. The amount of coffee fruit that is used in brewing coffee drinks varies significantly and is closely related to the method of brewing, the used type of coffee beans, and personal taste. Commonly, a cup of coffee requires 10 to 15 g of coffee powder, so at least 10 g of coffee powder for one coffee drink should be taken into consideration to gauge the health risk of coffee consumption. In other words, the weekly consumption of coffee beans should be around 40 g, which is 5.7 g/person/day. This value is considered a suitably conservative estimate that aligns with the actual habits of regular consumers in China, effectively covering the exposure levels of most regular drinkers while avoiding the overestimation of the risk that could result from an inflated consumption value. For the non-carcinogenic risk, the hazard index (HI) was calculated as follows:

$${ADD}_{nc}=\frac{C\times IngR\times EF\times ED\times CF}{BW\times {AT}_{nc}}$$

Non-carcinogenic risk:

$$HQ=\frac{ADD}{RfD}\;HI=\sum HQ$$

Carcinogenic risk:

LCR = ADD × SF TLTR = LCR

where ADD_nc_ and HQ are the chronic daily intakes from the ingestion pathways and target hazard quotients, respectively, while LCR and TLCR are used to denote the cancer risks of the ingestion pathways for a single element or all elements, respectively. All other relevant parameters for health risk assessment can be found in Table 4.

### 2.8. Statistical Analysis

The correlations between the data were analyzed by IBM SPSS Statistics (Version 20; IBM Corp., Armonk, NY, USA), and *p* < 0.05 indicated a significant difference in the data. Figures and tables were created with Origin Pro 8 and Coreldraw X4.

## 3. Results and Discussion

### 3.1. Content of Heavy Metals in Soil of Coffee-Growing Areas

The concentrations of heavy metals in the soils of the coffee plantations in Pu’er are presented in Table 5. In descending order, the mean concentrations followed the pattern Cr > Zn > Pb > Cu > As > Hg > Cd, with Cr being the most abundant element. With the exception of Cu and Zn, the concentrations of all heavy metals exceeded the corresponding background values for soils in Yunnan Province, indicating a notable enrichment effect in the region. However, the mean and maximum concentrations of most heavy metals remained well below the national risk screening values for agricultural land, suggesting overall acceptable soil quality. It is noteworthy that the maximum concentrations of Pb, Cr, and Cu at some sampling sites surpassed the risk screening values. This points to potential soil contamination in localized areas, which is of particular concern as these sites also exhibited significantly elevated levels of Pb and Cr relative to the background values.

### 3.2. Assessment of Heavy Metal Contamination in Soils of Coffee-Growing Areas

**Figure 2:**
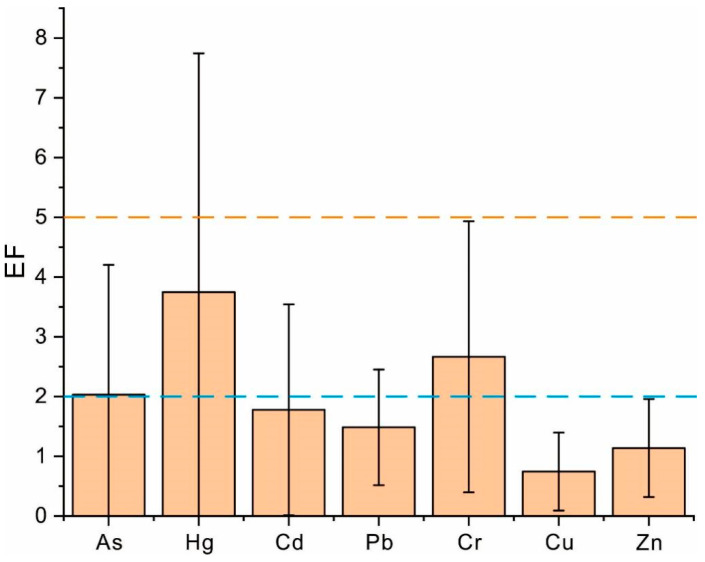
The enrichment of heavy metals in the soils of coffee-growing areas. **Note:** The blue and orange dash line are respectively the thresholds of no and moderate enrichment.

**Figure 3:**
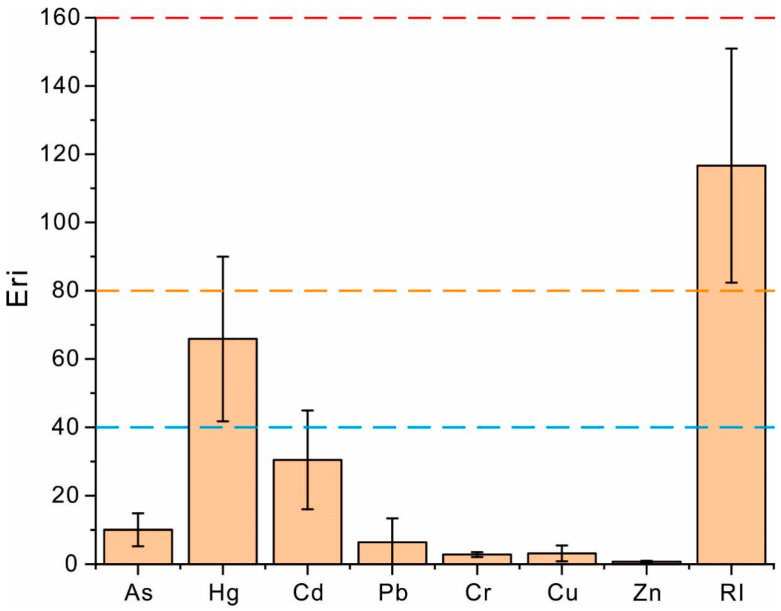
The monomial and total risk indices of heavy metals in the soils of coffee-growing areas. **Note:** The blue, orange and red dash line are respectively the thresholds of no, moderate and considerable pollution.

The enrichment factor (EF) and potential ecological risk index (RI) were introduced to evaluated heavy metal contamination in the soils of the coffee-planting areas in this study, the results of which are presented in Figure 2 and Figure 3. The EF values of heavy metals in the soils decreased in the order Hg > Cr > As > Cd > Pb > Zn > Cu. With the exception of Hg and Cr, the mean EF values of all heavy metals were below 2, indicating no enrichment and suggesting a minimal influence of anthropogenic activities. In contrast, both Hg and Cr exhibited significantly higher EF values, the mean values of which exceeded 2. For Hg, the maximum value of the EF even surpassed 5, which reflects a moderate to significant enrichment level at some sampling sites.

In terms of the potential ecological risk, the E_r_^i^ values of heavy metals followed the sequence Hg > Cd > As > Pb > Cu > Cr > Zn, with Hg showing the highest E_r_^i^ values. Overall, the RI values of heavy metals were all close to or below 150, indicating a generally low level of potential ecological risk from heavy metals in the soil. This is consistent with the fact that most E_r_^i^ values were well below 40. However, likely due to substantial exogenous input, the mean E_r_^i^ value of Hg significantly exceeded 40, even reaching up to 80 at certain sites, representing a moderate to considerable ecological hazard. Therefore, strengthening the control of exogenous Hg inputs is crucial in mitigating the potential ecological risks of heavy metals in the coffee-growing soils of Pu’er City.

### 3.3. Distribution of Heavy Metals in Coffee Plants

**Figure 4:**
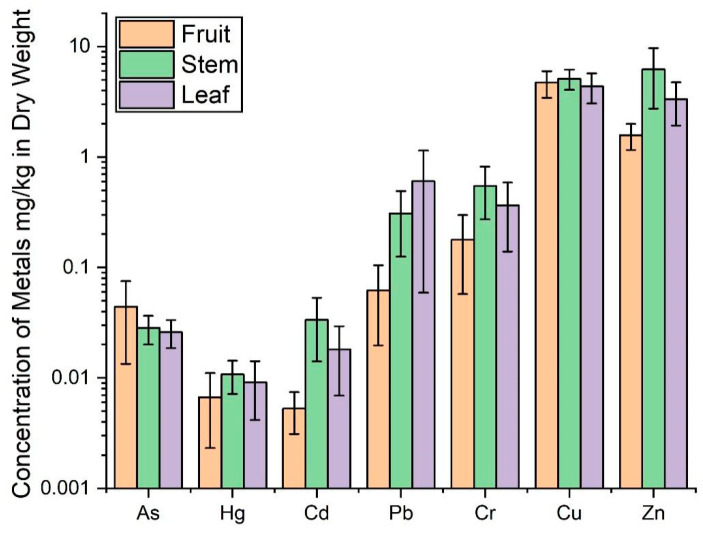
The distribution of heavy metals in different parts of coffee plants.

To determine the distribution of heavy metals in coffee plants, the concentrations in the fruit, stems, and leaves were analyzed (Table 6 and Figure 4). With the exception of Cr, the mean concentrations of heavy metals in fresh coffee beans were significantly lower than their corresponding maximum residue limits (MRLs), indicating relatively low contamination levels. In contrast, the concentration of Cr in the beans was approximately three times higher than its MRL, which is likely attributable to the elevated background levels of Cr in the local soils (Table 6). Therefore, controlling Cr accumulation in the soil is crucial in improving coffee quality. Overall, the heavy metal concentrations in coffee plants decreased in the order of Cu > Zn > Cr > Pb > As > Cd > Hg. Unlike in the soil, where Cr was the most abundant, Cu showed the highest concentration in the plants, suggesting the stronger bioconversion of Cu. An analysis of different plant parts revealed that the stems contained the highest concentrations of most heavy metals, except for As and Pb. This distribution pattern can be explained by the transport dynamics of heavy metals: they are absorbed by the roots and transported upward through the xylem with the transpiration stream [56]. During this process, metals are often immobilized or retained by the xylem walls in the stems [2], leading to their accumulation. Moreover, the self-protection mechanism of plants, involving the phloem barrier, actively restricts the secondary translocation of heavy metals to the leaves and fruits, further contributing to their preferential accumulation in the stems [56,57].

However, not all heavy metals accumulate predominantly in the stems; the leaves and fruits also exhibit significant potential for metal enrichment. In the case of leaves, their role as primary “metabolic factories” and the strong transpirational pull that they generate serve as the major driving forces for the upward transport of metal ions via the xylem [58,59]. Once delivered to the leaves, heavy metals are often chelated by intracellular proteins and organic acids, leading to their sequestration and accumulation [60,61]. Pb, in particular, shows a distinct tendency for leaf enrichment due to three synergistic mechanisms: firstly, its low solubility and mobility limit its redistribution from the leaves after xylem transport [62], causing retention; secondly, Pb readily forms insoluble compounds with cell wall components and phosphates [63], becoming further immobilized in the apoplast or vacuoles; and, thirdly, as a direct interface with the environment, the leaves can absorb atmospherically deposited Pb through the stomata or the epidermis, providing an additional pathway beyond root uptake [64]. These three processes collectively explain the relative enrichment of Pb in the leaves compared to other plant parts (Figure 4).

In the case of coffee fruits, the concentration of As was notably higher compared to other plant parts, whereas the levels of other heavy metals were generally lower than those in the leaves and stems (Figure 4). This indicates a relatively limited capacity for the accumulation of most heavy metals in coffee berries, suggesting that the edible fruit is less affected by heavy metal contamination in soil. Indeed, as the fruits and seeds are crucial for plant reproduction, plants have evolved a self-protective mechanism known as the “fruit barrier” to minimize the transfer of contaminants into these reproductive organs [65]. Previous studies have shown that plants employ chelators such as phytochelatins (PCs) and metallothioneins (MTs) to bind heavy metal ions, which are then compartmentalized within vacuoles or other organelles [37,66]. This process inactivates the metals and restricts their translocation to the fruits, thereby protecting the embryo and ensuring seed viability and reproductive success. This mechanism explains the generally low concentrations of most heavy metals reported in fruits.

While the “fruit barrier” effectively reduces the accumulation of many contaminants, its efficacy is largely confined to cationic metals. For certain pollutants with an anionic nature, such as arsenic (As), the barrier’s mitigating effect is diminished. Arsenic, a metalloid, can exist in anionic forms—e.g., arsenite (As(III)) and arsenate (As(V))—under typical soil conditions [67,68]. These anionic species are less effectively chelated by PCs and MTs [69]. Consequently, As is often taken up and transported alongside essential nutrient elements such as phosphorus and silicon [70], enabling it to bypass the fruit barrier and accumulate more readily in coffee beans. Although the absolute concentration of As in coffee fruits reported in this study was not exceptionally high, its relative enrichment compared to other plant tissues warrants attention. Given the well-established carcinogenicity of arsenic [71], the observed accumulation trend in coffee berries deserves consideration and may have important implications for the quality and safety of coffee.

**Table 6:** Heavy metal content in coffee fruit and their maximal residual limits, mg/kg dry weight.

Fruit	As	Hg	Cd	Pb	Cr	Cu	Zn
Min–Max	0.01–0.13	N.D.–0.02	N.D.–0.01	0.02–0.16	0.05–0.38	3.06–7.43	1.02–2.35
Mean	0.04	0.007	0.01	0.06	0.18	4.71	1.57
MRL	-	0.05 ^a^	0.3 ^b^	0.10 ^c^	0.05 ^d^	-	-

Note: MRL is the maximal residual limit. The referenced standards are as follows: ^a^ maximum level for As in instant coffee according to China National Food Safety Standard (GB 2762-2022) [72]; ^b^ maximum level for Pb in coffee beans according to European Commission Regulation (EC) No. 1881/2006 [73]; ^c^ maximum level for Cd in coffee according to Codex Alimentarius Commission (CODEX STAN 193-1995) [74]; ^d^ maximum level for As according to Japan’s Food Sanitation Act [75].

### 3.4. Bioconversion of Heavy Metals Within Soil–Coffee System

**Figure 5:**
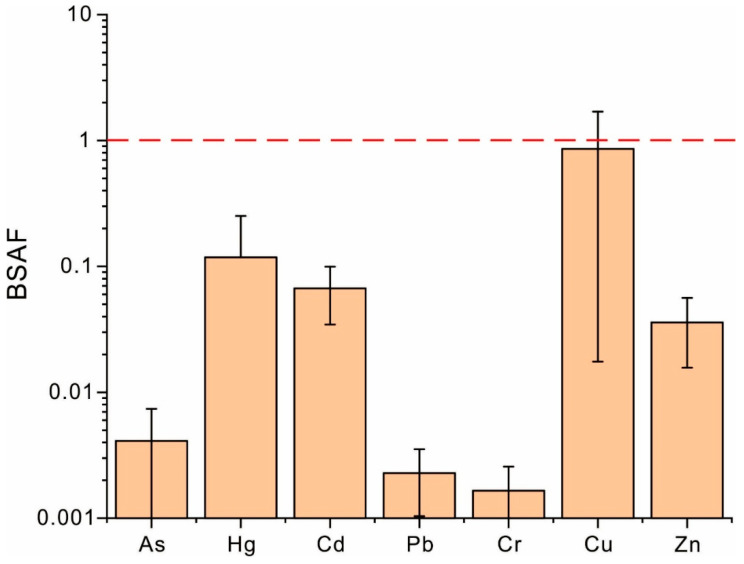
The BSAF values of heavy metals within the soil–coffee system. **Note:** The red dash line is the threshold of BSAF.

To elucidate the bioconversion of heavy metals within the soil–coffee system, the BSAF values of heavy metals were calculated, with results presented in Figure 5. The mean values of the BSAF for heavy metals decreased in the order Cu > Hg > Cd > Zn > As > Pb > Cr. Although the average BSAF value of Cu did not exceed 1, the maximal BSAF value of Cu, which was significantly beyond 1, suggests relatively high potential for bioconversion and a tendency for Cu to accumulate in coffee plants, particularly in some sampling sites. In contrast, the BSAF values of other heavy metals were all substantially below 1, suggesting generally low bioconversion and a tendency for these metals to be retained in the soil, thereby posing minimal impacts on coffee quality. To further identify interactions between soil and coffee, the correlations between the content of heavy metals in the soil and their BSAF values were analyzed and are given in Table 7. While it is commonly reported that higher content of heavy metals in soil enhances their bioconversion [76], this study only observed a significant positive correlation between the concentrations of Cr in the soil and their BSAF values (Table 7), indicating that the effect of the total content of heavy metals in soil on their bioconversion should be limited. This limited influence can be attributed to two main mechanisms. First, plants possess various physiological defense mechanisms that restrict the uptake of heavy metals under their stresses [37]. As the stresses caused by heavy metals are intensified as their concentrations in the soil increase, plants enhance their defensive capabilities to limit the absorption of heavy metals, thereby suppressing the bioconversion of heavy metals [66], which is directly expressed as the weakening of the positive relationship between the heavy metal content and their BSAF values. Second, heavy metals in weakly bound fractions, which are critical for the bioavailability of heavy metals, typically constitute a very small portion (often <5%) of the total content of heavy metals in the soil [36,77,78,79]. This fraction would probably not increase proportionally with an increase in the total concentration of heavy metals, which constrains the impact of the elevated total content of heavy metals in terms of enhancing their bioconversion. Although this study did not monitor plant physiological processes or analyze the speciation of the heavy metals in soils, the significant negative correlations observed between the content of Hg, Pb, Cu, and Zn in the soil and their BSAFs could still support the conclusion that the overall influence of the total concentration of heavy metals in soil on their bioconversion should be predominantly negative rather than positive.

### 3.5. Health Risk Assessment of Heavy Metals in Coffee

**Figure 6:**
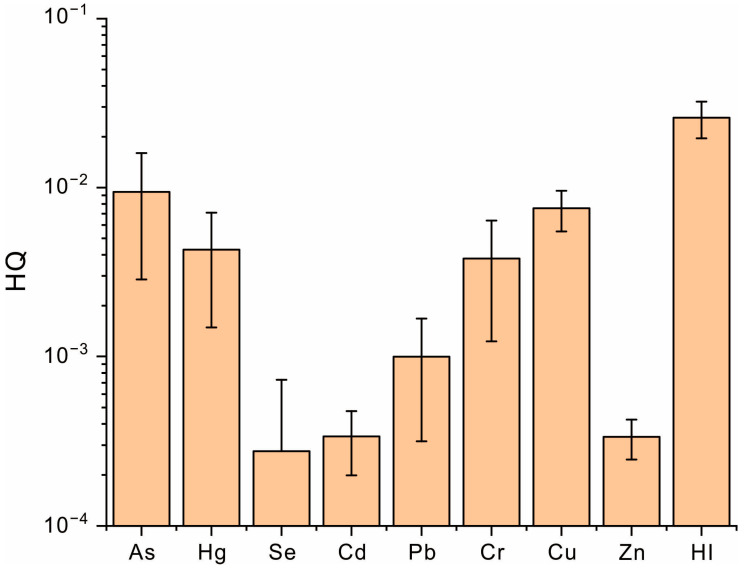
The non-carcinogenic risks of heavy metals in coffee fruit.

**Figure 7:**
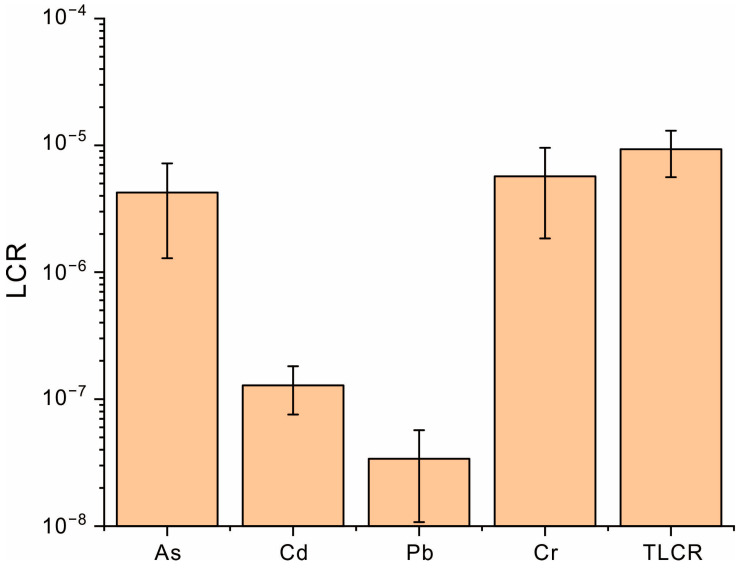
The carcinogenic risks of heavy metals in coffee fruit.

To assess the health risks of coffee consumption associated with heavy metals in Pu’er City, this study calculated both the non-carcinogenic and carcinogenic risks posed by heavy metals, with the results displayed in Figure 6 and Figure 7. For the non-carcinogenic risk, the HQ values of heavy metals decreased in the order As > Cu > Hg > Cr > Pb > Zn > Cd. As showed the highest HQ value, identifying it as the primary contributor to the non-carcinogenic risk for coffee consumption. Although As posed the highest non-carcinogenic risk among all heavy metals, its HQ value remained below 1, indicating a negligible risk of non-carcinogenic effects from As exposure through coffee consumption. Overall, both the individual HQ value and HI value were well below the threshold of 1, suggesting no significant non-carcinogenic health risk from drinking coffee produced in this area. In terms of the carcinogenic risk, the LCR values decreased in the sequence Cr > As > Cd > Pb. Cr exhibited the highest LCR value, establishing it as the dominant carcinogenic risk factor. The LCR value for Cr exceeded 1 × 10^−6^, indicating a detectable carcinogenic risk from excessive exposure; however, it remained substantially below 1 × 10^−4^, suggesting that the risk is generally acceptable. Similarly, the LCR value for As also fell within the range of 1 × 10^−6^ to 1 × 10^−4^, implying a potential but acceptable carcinogenic risk. In contrast, the LCR values for Cd and Pb were significantly lower than 1 × 10^−4^, indicating that coffee consumption is unlikely to lead to appreciable carcinogenic risks from these heavy metals. Collectively, the TLCR exceeded 1 × 10^−6^ due to the combined effects of Cr and As. It can be concluded that heavy metals in coffee may pose a measurable, albeit overall low and acceptable, carcinogenic risk. To further minimize this risk, it is crucial to control the bioavailability of As and Cr and then lower their bioconversion within the soil–coffee system.

## 4. Conclusions

This study provides a comprehensive assessment of the heavy metals in the soil–coffee system of Pu’er City. The findings highlight Hg as the primary pollutant of ecological concern, while As and Cr contribute to a low but detectable carcinogenic risk through coffee consumption. To ensure the sustainable and safe development of the local coffee industry, the following measures are recommended: (1) implementing targeted source control measures to mitigate the input of Hg and Cr, potentially through the regulation of fertilizers and pesticides; (2) investigating the specific mechanisms governing the relatively higher transfer of As into coffee beans to develop targeted mitigation strategies; and (3) promoting agricultural practices such as soil liming to reduce the bioavailability of heavy metals in these acidic soils. Integrating these findings into regional environmental zoning and risk management frameworks is crucial in safeguarding both ecosystem health and the market reputation of Pu’er’s coffee.

It should be noted that this study measured the total heavy metal concentrations in the soils and plants, which is standard for environmental risk assessment. However, the water-soluble forms of metals may be more relevant regarding bioavailability in coffee beverages. Future studies should include speciation analysis to better understand the transfer of heavy metals into the beverage.

## Figures and Tables

**Table 1:** The classification of heavy metal contamination [43,44,45].

Class	EF	Level of Enrichment
1	0.5 < EF < 2	No Enrichment
2	2 ≤ EF < 5	Moderate Enrichment
3	5 ≤ EF < 20	Significant Enrichment
4	20 ≤ EF < 40	Severe Enrichment
5	EF < 40	Extremely Severe Enrichment

**Table 2:** Soil background values and toxicity factors of HMs [43,44,49].

Item	Cr	Cu	Zn	Pb	Cd	As	Hg
$C_{n}^{i}$ (mg/kg)	69.6	37.6	88.8	36.5	0.08	14.9	0.05
$T_{r}^{i}$	2	5	1	5	30	10	40

**Table 3:** The classification of heavy metals’ ecological risks [43,44,45].

Grade	E_r_^i^	Ecological Risk Level
1	<40	Low
2	40–80	Moderate
3	80–160	Considerable
4	160–320	High
5	>320	Very high
Grade	RI	Ecological risk level
1	<150	Low
2	150–300	Moderate
3	300–600	Considerable
4	>600	Very high

**Table 4:** Values of variables for estimation of human health risk.

Parameter		Unit	Definition		Value		Reference	
C		µg kg^−1^	Metal content		**-**		-	
BW		kg	Average body weight		70		[16]	
ED		year	Exposure duration		30		[52]	
EF		d yr^−1^	Exposure frequency		350		[52]	
IngR		g d^−1^	Ingestion rate		5			
AT_nc_		d	Averaging times for non-carcinogenic effects		9125		[17]	
AT_ca_		d	Averaging times for carcinogenic effects		26,280		[17]	
CF		kg mg^−1^	Conversion factor		10^−6^		[18]	
Ingestion	Metal	Pb	Cd	Cr	Hg	As	Zn	Cu
	RfD	3.50 × 10^−3^	1.00 × 10^−3^	3.00 × 10^−3^	3.00 × 10^−4^	3.00 × 10^−4^	3.00 × 10^−1^	4.00 × 10^−2^
	SF	8.50 × 10^−3^	6.10 × 10^0^	5.01 × 10^−1^	-	1.50 × 10^0^	-	-

Note: RfD and SF are the oral reference dose and oral slope factor (mg/kg/d), respectively, both of which can be found in a previous study by Miao, Miao, Hao, Xie, and Zou [13].

**Table 5:** Heavy metal content in soils of coffee-growing areas and other baselines, mg/kg dry weight.

		As	Hg	Cd	Pb	Cr	Cu	Zn
Pu’er City	Min–Max	3.41–30.65	0.02–0.17	0.03–0.17	9.89–193	65.1–157	1.32–60.2	14.8–118
	Mean	14.96	0.08	0.08	46.97	98.47	23.68	62.50
Soil Baseline for Yunnan Province [7]	Median	13	0.0875	0.07	22.35	88.7	24.75	63.2
Risk Screening Value for Agricultural Land [55]		14.9	0.05	0.08	36.5	69.6	37.6	88.8

**Table 7:** The correlations of the heavy metal content in the soil and their BSAF values.

BSAF	As	Hg	Cd	Pb	Cr	Cu	Zn
Content in soil	−0.467	−0.875 **	−0.381	−0.551 *	0.755 **	−0.686 **	−0.891 **

** Correlation is significant at 0.01 level (2-tailed); * correlation is significant at 0.05 level (2-tailed).

## Data Availability

The raw data supporting the conclusions of this article will be made available by the authors on request.
